# Aurora Kinase A expression predicts platinum-resistance and adverse outcome in high-grade serous ovarian carcinoma patients

**DOI:** 10.1186/s13048-016-0238-7

**Published:** 2016-05-21

**Authors:** Chiara Mignogna, Nicoletta Staropoli, Cirino Botta, Carmela De Marco, Antonia Rizzuto, Michele Morelli, Annalisa Di Cello, Renato Franco, Caterina Camastra, Ivan Presta, Natalia Malara, Angela Salvino, Pierfrancesco Tassone, Pierosandro Tagliaferri, Tullio Barni, Giuseppe Donato, Anna Di Vito

**Affiliations:** Department of Health Science, Pathology Unit, Magna Græcia University of Catanzaro, Medical School, Viale Europa, 88100 Catanzaro, Italy; Department of Experimental and Clinical Medicine, Medical Oncology, Magna Græcia University of Catanzaro, Medical School, Viale Europa, 88100 Catanzaro, Italy; Translational Medical Oncology Unit, Department of Experimental and Clinical Medicine, Magna Græcia University, Catanzaro, Italy; Department of Experimental and Clinical Medicine, Magna Græcia University, Catanzaro, Italy; Department of Medical and Surgical Sciences, Magna Græcia University, Catanzaro, Italy; Department of Mental and Physical Health and Preventive Medicine, Second University of Naples, Naples, Italy

**Keywords:** High-grade serous ovarian carcinoma (HGSOC), AURKA, Platinum, Therapy, Prognosis

## Abstract

High-Grade Serous Ovarian Carcinoma (HGSOC) is the predominant histotype of epithelial ovarian cancer (EOC), characterized by advanced stage at diagnosis, frequent TP53 mutation, rapid progression, and high responsiveness to platinum-based-chemotherapy. To date, standard first-line-chemotherapy in advanced EOC includes platinum salts and paclitaxel with or without bevacizumab. The major prognostic factor is the response duration from the end of the platinum-based treatment (platinum-free interval) and about 10–0 % of EOC patients bear a platinum-refractory disease or develop early resistance (platinum-free interval shorter than 6 months). On these bases, a careful selection of patients who could benefit from chemotherapy is recommended to avoid unnecessary side effects and for a better disease outcome. In this retrospective study, an immunohistochemical evaluation of Aurora Kinase A (AURKA) was performed on 41 cases of HGSOC according to platinum-status. Taking into account the number and intensity of AURKA positive cells we built a predictive score able to discriminate with high accuracy platinum-sensitive patients from platinum-resistant patients (*p* < 0.001). Furthermore, we observed that AURKA overexpression correlates to worse overall survival (*p* = 0.001; HR 0.14). We here suggest AURKA as new effective tool to predict the biological behavior of HGSOC. Particularly, our results indicate that AURKA has a role both as predictor of platinum-resistance and as prognostic factor, that deserves further investigation in prospective clinical trials. Indeed, in the era of personalized medicine, AURKA could assist the clinicians in selecting the best treatment and represent, at the same time, a promising new therapeutic target in EOC treatment.

**Electronic supplementary material:**

The online version of this article (doi:10.1186/s13048-016-0238-7) contains supplementary material, which is available to authorized users.

## Background

Epithelial ovarian cancer (EOC) represents the 5th leading cause of cancer-related death worldwide [1] with approximately 225.500 new diagnoses *per* year and a mortality rate greater than 30 % [2]. High-Grade Serous Ovarian Carcinoma (HGSOC) is the most aggressive histotype, and accounts for 60–80 % of all ovarian carcinoma [3, 4]. Particularly, HGSOC is characterized by rapid progression and frequent TP53 mutations [5–7]. Primary treatment for HGSOC includes surgery and platinum/taxane based chemotherapy. However, even though 70–80 % of patients show an initial response to chemotherapy, approximately 25 % relapse within 6 months [8, 9].

According to time to relapse after last chemotherapy administration, EOC patients are classified into three platinum-status groups. Patients who experience a recurrence after 6 months are deemed platinum-sensitive (PS) and are good candidates for a platinum rechallenge [10]. Conversely, patients who relapse within 6 months are classified as platinum-resistant (PR), and are candidate to alternative treatment schedules that do not include platinum-derived compounds [11, 12]. Approximately 20 % of all EOC patients belong to this latter group [13]. Lastly, the platinum-refractory group involves patients who experience disease progression during the course of treatment. This is the subgroup with the worse prognosis and includes less than 10 % of HGSOC patients [14].

The molecular basis of platinum-resistance is not yet fully understood and experimental results suggest the involvement of several cellular functions, such as: changes in cellular uptake and efflux of cisplatin, increased biotransformation and detoxification in the liver, loss of apoptotic signaling after DNA damage has occurred, DNA repair or DNA damage tolerance. Specifically, genes previously implicated in EOC pathogenesis such as AURKA1, ERBB3, CDK2, and mTOR, and novel candidates such as BRD4, VRK1 and GALK1, have been shown to be involved in such features, thus becoming potential predictive/prognostic markers in HGSOC [15]. In addition, HDAC4, STAT1, FORL2 and PIK3R1 were over-expressed in resistant cells when compared to sensitive cells, suggesting their functional involvement in platinum-resistance [16].

Recently, a meta-analysis indicated *Aurora kinase A* (AURKA) as an effective prognosticator in solid tumors patients, including EOC [17]. Accordingly, a number of new AURKA inhibitors, such as ZM447439, Hesperadin, VX-680/MK-0457, AT9283 and AZD1152 are being developed to target malignant tumors and clinical trials are ongoing to investigate their efficacy [18].

Aurora kinases are a family of serine/threonine kinases that play a critical role in the regulation of mitosis, especially in the distribution of genetic material to daughter cells [19]. In particular, AURKA has been extensively investigated for its implication in different neoplasms [20] and it has been identified as a low penetrance tumor-susceptibility-gene in human cancer [21]. Moreover, specific AURKA polymorphisms have been associated with carcinogenesis [22–28], while its overexpression has been described in various types of tumors, including laryngeal, breast, colon, pancreas, ovarian, bladder, liver, and gastric cancers [29]. This condition may derive from gene amplification as well as deregulation of gene expression; in addition, inhibition of protein degradation was also reported [20, 30]. The molecular mechanism by which AURKA contributes to tumorigenesis is complex, often implying apoptosis and/or autophagy signaling perturbation. Indeed, AURKA interacts with many tumor suppressor proteins (p53, BRCA1, glycogen synthase kinase (GSK)-3b, and c-Myc), thus accounting for significant alteration of their modulatory functions [31–34]. Furthermore, AURKA overexpression seems to occur as an early event in EOC development [35, 36].

On these bases, we investigated the association between the expression of AURKA in HGSOC patients’ specimens and clinical outcome, taking into account both response to chemotherapy and survival.

## Methods

Forty-one patients with confirmed diagnosis of HGSOC (mean age: 63.43 years; range: 44–88 years) consecutively treated in our institution between 2009 and 2015, were enrolled in this study. Eligibility criteria included histological diagnosis of EOC, age > 18 years old, at least one previous line of treatment including a platinum-based schedule. Patients were grouped as PR (platinum-resistant) and PS (platinum-sensitive) taking into account the classification-system based on the “platinum-free interval”, i.e. the time-frame between the end of chemotherapy treatment and recurrence, as previously described.

Surgical specimens were retrieved from the archive files of the Department of Health Sciences, Surgical Pathology Section of the University Magna Græcia of Catanzaro, Italy. Clinical data have been retrieved from medical records of the Gynecologic and Medical Oncology Units of the same Institution. All data were entered into an electronic database.

A detailed clinical follow-up, ranging from 3 to 50 months after surgery (average value: 19.13 months) was available for all patients. Written informed consent was obtained from each patient being studied and all procedures performed in this study were performed for diagnostic purposes, in accordance with the ethical standards of the institutional committee responsible for human experimentation.

Immunohistochemical staining procedures were carried out on formalin-fixed, paraffin-embedded archival cell blocks. For AURKA identification, a three layer biotin-avidin-peroxidase system was utilized. Briefly, xylene dewaxed and alcohol-rehydrated serial tissue sections (4 μm-thick) were treated in EDTA buffer at 98 C° for 50 min, according to the antigen retrieval method. Afterwards, endogenous peroxidase activity was blocked with 3 % hydrogen peroxide solution. For the evaluation of AURKA signal, mouse monoclonal anti-Aurora Kinase 2 (clone JLM28, 1:50 dilution; Leica Biosystems, United Kingdom) was applied for 1 h at RT. Biotinylated secondary antibody and avidin-biotin peroxidase complex were then applied and allowed to react for 30 min at RT. AURKA signals were visualized after addition of 0.01 % DAB (3, 3’-diaminobenzidine tetrahydrochloride). Nuclear counterstaining was perfomed with hematoxylin.

**Fig. 1:**
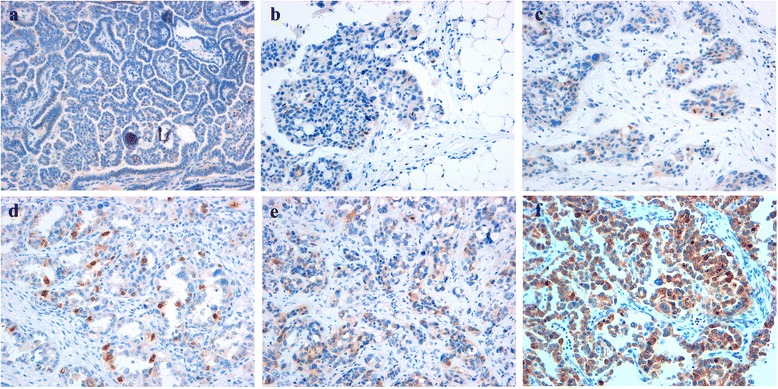
Immunohistochemical analysis of AURKA in surgical specimens of HGSOC (percentage-based approach). The panel shows a proportion score assigned on the basis of the percentage of AURKA positive tumor cells. 0: no AURKA-positive cells (**a**), 1:<1 % (**b**), 2: 1–10 % (**c**), 3: 10–33 % (**d**) 4: 33–66 % (**e**), 5: 66–100 % (**f**). Magnification 100x (**a**), 200x (**b**-**f**)

**Fig. 2:**
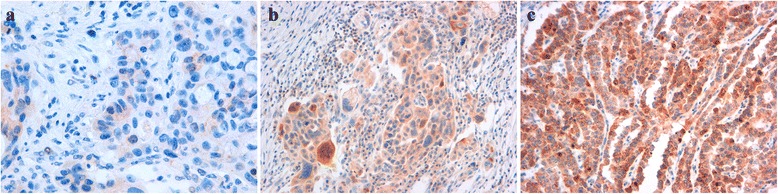
Immunohistochemical analysis of AURKA in surgical specimens of HGSOC (intensity-based approach). The panel shows three examples of different intensity score value**.** 1 = weak (**a**); 2 = intermediate (**b**); 3 = strong (**c**) Magnification 400x (**a**), 200x (**b**, **c**)

A semi-quantitative analysis was performed, evaluating both percentage of AURKA-positive cells and staining intensity (intensity and percentage-based approaches), using the score system by Allred et al. [37–39]. A percentage-based approach was used in order to estimate the proportion of positively stained tumor cells (0: none, 1:<1 %, 2: 1–10 %, 3: 10–33 % 4: 33–66 %, 5: 66–100 %) Fig. 1 (a-f). Average estimated intensity of staining in positive cells was assigned as an intensity score (0 = none; 1 = weak; 2 = intermediate; 3 = strong) Fig. 2 (a-c).

An immunoreactive score ranging from 0 to 8 was defined as the sum of percentage score and intensity score. Negative control sections for immunohistochemistry were processed without primary antibody.

Two investigators performed clinical data collection and statistical analyses independently.

Primary endpoint was response rate (RR) according to platinum response. Secondary endpoint was overall survival (OS) in all patients. A Student's test was used to compare the scores of the two patient groups. Kaplan Meier curves and Log Rank test were used to describe and evaluate the effect of several variables on outcome. All tests were considered statistically significant when the p-value was <0.05. The relative hazard ratios (HR) with 95 % confidence intervals (95 % CIs) were calculated using SPSS (version 19) statistical package and Graphpad PRISMA (version 6.0).

## Results

Clinical and pathological parameters of patients and immunohistochemical findings are detailed in Table 1. To verify the reliability of the sample, we evaluated if patients with a platinum-sensitive disease experienced the longest survival. As reported in Additional file 1: Figure S1, according to published literature, patients in the PS group presented the best outcome (50 versus 14 months, *p* < 0.0001) thus confirming the reliability of the sample. Subsequently, we confirmed the original diagnosis of HGSOC for all cases examined. Two cases were stage IIA, 1 case stage IIB, 4 cases stage IIC, 1 case stage IIIA, 7 cases stage IIIB, 16 cases stage IIIC and 10 cases stage IV tumours according to AJCC guidelines.

**Table 1:**
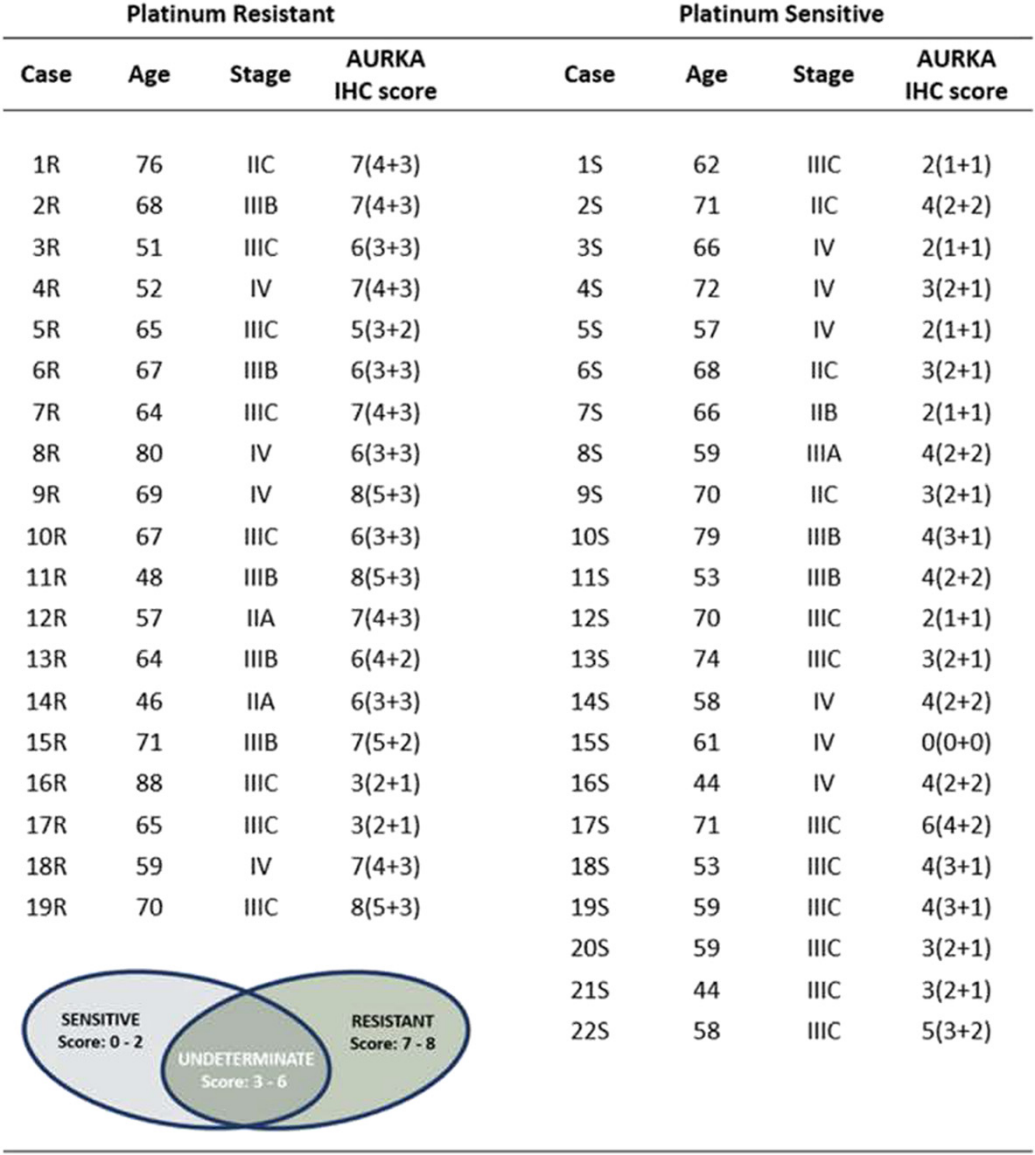
Distribution of AURKA in HGSOC patients. The average score value of AURKA is low in platinum sensitive patients and high in platinum resistant patients ***(*p* < 0.001)

**Fig. 3:**
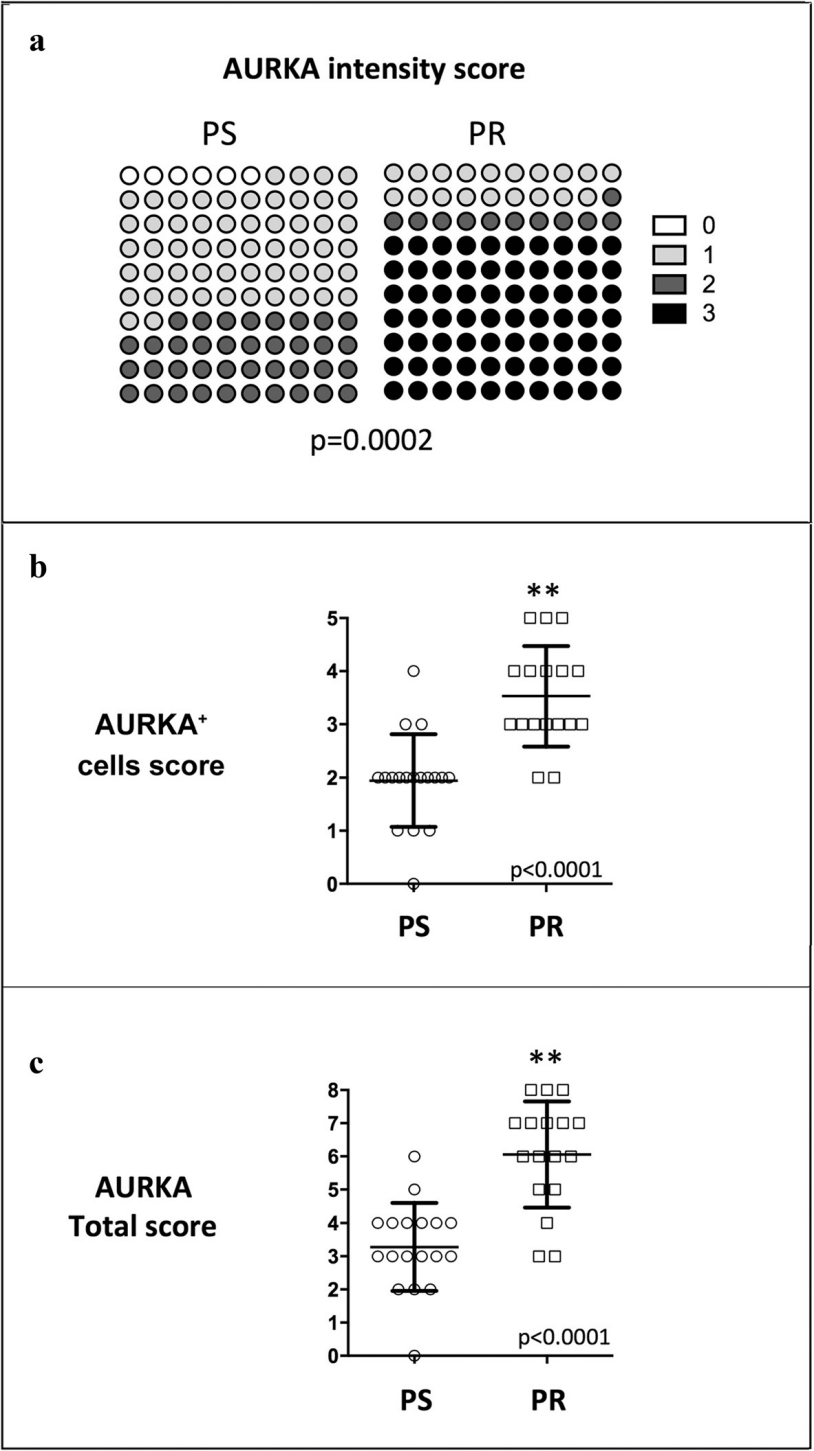
Correlation of AURKA scores with platinum-response. A strong association between both Intensity (Panel **a**) and Cell percentage (Panel **b**) scores with platinum response is showed. Panel **c** reports the correlation between AURKA total score and platinum response

The expression of AURKA in neoplastic cells, using the specific score system, was comprised between 0 and 8. Figure 3 shows the average score value described for each group of patients.

As shown in Fig. 3a, both AURKA intensity score and AURKA^+^ cells score were highly associated with platinum-response. However, the AURKA total score demonstrated the highest capability to discriminate patients belonging to the two different platinum-sensitivity conditions (*p* < 0.001).

**Fig. 4:**
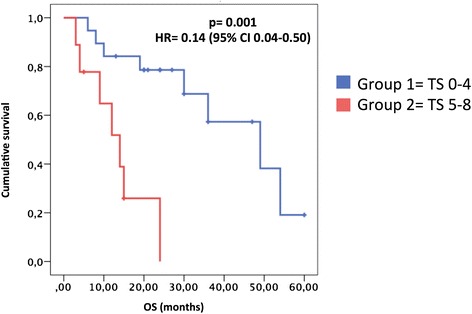
Correlation of AURKA scores with survival. Kaplan Meier curves of EOC patients grouped according to high or low AURKA total score. TS: Total Score

Furthermore, high levels of AURKA were also correlated to a worse overall survival (*p* = 0.001; HR 0.14) (Fig. 4).

## Discussion

At present, platinum-based regimens represent the best therapeutic option for EOC patients. Taking into account that platinum-response is one of the most important prognostic factors for these patients, the identification of predictive biomarkers may be crucial in clinical practice.

In our study, we observed a statistically significant correlation between high level of AURKA in HGSOC specimens and platinum-resistance. Our results are in line with *in vitro* studies, demonstrating that overexpression of AURKA accounts for resistance to taxane and platinum agents [17, 18, 40, 41]. Additionally, recent reports show that cells depleted of AURKA are more sensitive to cisplatin-induced apoptosis, and elevated expression of Aurora kinase A antagonizes this response [42].

From a practical point of view, our series may be stratified into three different groups according to the score obtained and the platinum-status. All the patients with scores 6–8 were platinum-resistant, while those with scores 0–2 were all platinum-sensitive. Therefore, we have determined two cut-off (score 2 and score 6) that seem to be able to identify the patients responsiveness to platinum treatment from chemoresistant patients. Patients with intermediate score, between three and six presented contrasting outcomes. For these patients, a FISH-based approach or further molecular analyses should be recommended in order to deeply characterize and quantify the expression of AURKA and subsequently correlate it with outcome measures.

Taking into account the poor prognosis of platinum-resistant patients, the routine use of this new biomarker could help clinicians in choosing the best frontline treatment. Indeed, by evaluating the AURKA pattern, chemoresistant patients in advanced stages could be easily identified, in order to prevent unnecessary severe side effects as well as to reduce treatment costs [41].

To the best of our knowledge, the expression and prognostic significance of AURKA in HGSOC has been poorly investigated. In 2007, Lessmann et al. suggested AURKA as a predictive marker in EOC. However, no data have been yet reported on its potential role in the prediction of response to platinum/taxane treatment [43].

The major limitations of our study is the low number of patients enrolled, the retrospective design and the lack of an independent validation series. Indeed, our work should be considered “hypothesis generating” and further prospective validation is eagerly awaited and is planned in the next future.

## Conclusion

The reported findings suggest AURKA as a new tool to predict the clinical behavior of HGSOC. Particularly, these results suggest that AURKA may have a role both as predictor of platinum-resistance and prognostic factor. Indeed, in the era of personalized medicine, the availability of new predictive biomarkers is crucial for the selection of better treatment in the scenario of a continuum of care. Moreover our findings suggest that targeting AURKA may hold promise as a new therapeutic strategy in EOC management.

## Declaration

The authors declare that the work described has not been published previously.

## Ethics approval and consent to participate

Not applicable.

## Consent for publication

Informed consents to participate in the study were obtained from participants.

## Availability of data and material

The datasets supporting the conclusions of this article are included within the article.

## Additional file

Additional file 1:
**Figure S1**. Correlation of platinum sensitivity status with survival. Kaplan Meier curves of EOC patients grouped according to platinum sensitive (PS) or resistant (PR) disease. Patients with a platinum sensitive ovarian cancer presents a significant longer survival thus confirming the reliability of the sample. (JPG 182 kb)

## Abbreviations

AURKA: Aurora Kinase A
DAB: 3, 3’-diaminobenzidine tetrahydrochloride
EOC: epithelial ovarian cancer
HGSOC: High-Grade Serous Ovarian Carcinoma
HR: hazard ratios
OS: overall survival
PR: platinum-resistant
PS: platinum-sensitive
RR: response rate
