# Supplementary figures and images for: Aurora Kinase A expression predicts platinum-resistance and adverse outcome in high-grade serous ovarian carcinoma patients
Source: J Ovarian Res. 2016 May 21;9:31. doi: 10.1186/s13048-016-0238-7 (PMC4875597; doi:10.1186/s13048-016-0238-7)

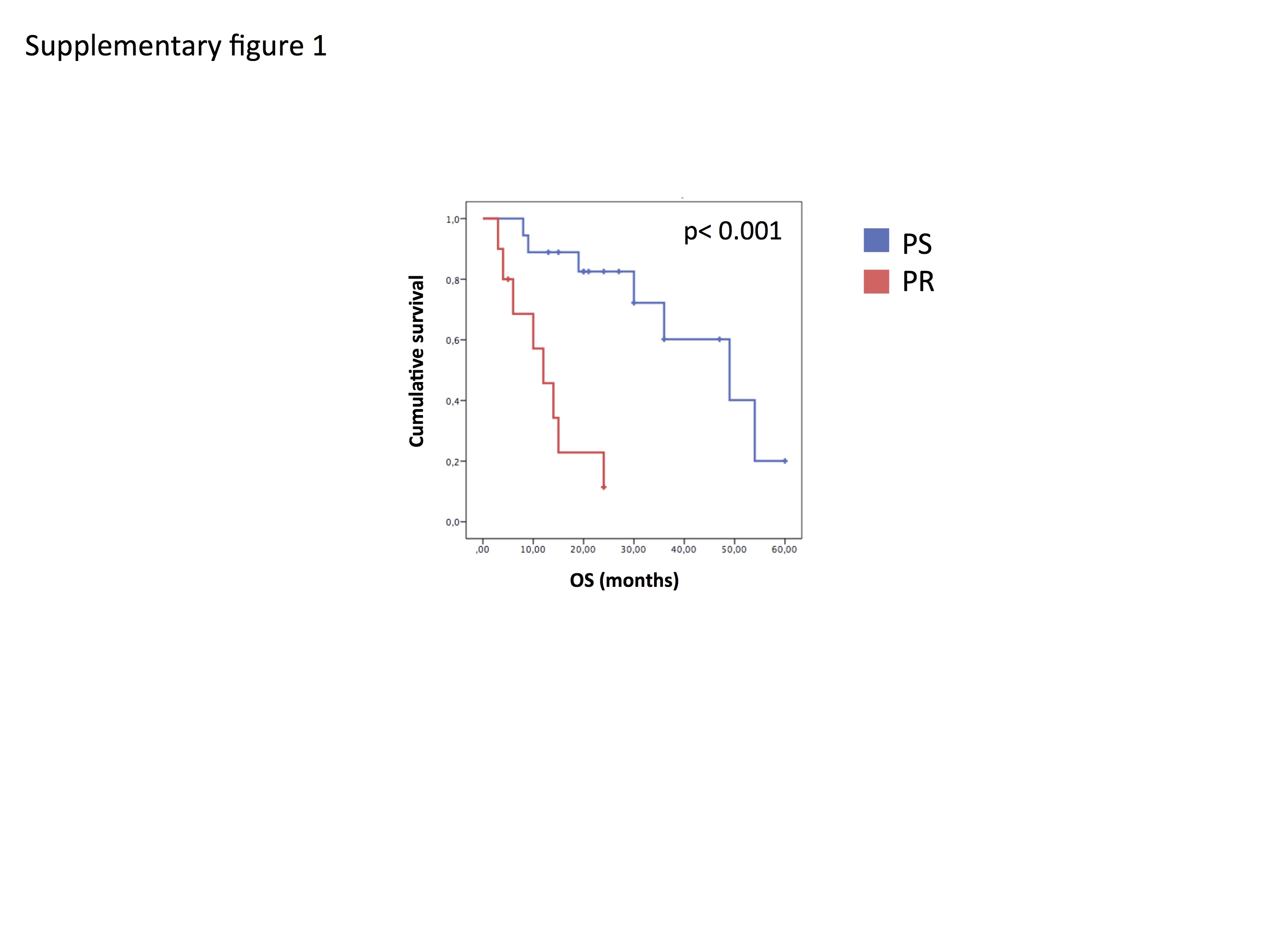

Supplement: Additional file 1: — Figure S1. Correlation of platinum sensitivity status with survival. Kaplan Meier curves of EOC patients grouped according to platinum sensitive (PS) or resistant (PR) disease. Patients with a platinum sensitive ovarian cancer presents a significant longer survival thus confirming the reliability of the sample. (JPG 182 kb) [file 13048_2016_238_MOESM1_ESM.jpg]
